# Angiotensin-I-Converting Enzyme (ACE) Inhibitors from Marine Resources: Prospects in the Pharmaceutical Industry

**DOI:** 10.3390/md8041080

**Published:** 2010-03-31

**Authors:** Isuru Wijesekara, Se-Kwon Kim

**Affiliations:** 1 Marine Biochemistry Laboratory, Department of Chemistry, Pukyong National University, Busan 608-737, Korea; E-Mail: isurumatara@yahoo.com (I.W.); 2 Marine Bioprocess Research Center, Pukyong National University, Busan 608-737, Korea

**Keywords:** angiotensin-I-converting enzyme inhibitors, antihypertensive activity, bioactive peptides, chitooligosaccharides, hypertension, phlorotannins

## Abstract

Hypertension or high blood pressure is one of the major independent risk factors for cardiovascular diseases. Angiotensin-I-converting enzyme (EC 3.4.15.1; ACE) plays an important physiological role in regulation of blood pressure by converting angiotensin I to angiotensin II, a potent vasoconstrictor. Therefore, the inhibition of ACE activity is a major target in the prevention of hypertension. Recently, the search for natural ACE inhibitors as alternatives to synthetic drugs is of great interest to prevent several side effects and a number of novel compounds such as bioactive peptides, chitooligosaccharide derivatives (COS) and phlorotannins have been derived from marine organisms as potential ACE inhibitors. These inhibitory derivatives can be developed as nutraceuticals and pharmaceuticals with potential to prevent hypertension. Hence, the aim of this review is to discuss the marine-derived ACE inhibitors and their future prospects as novel therapeutic drug candidates for treat hypertension.

## 1. Introduction

Hypertension or high blood pressure is one of the major independent risk factors for cardiovascular diseases [1,2] and it is a major health issue, estimated to be affecting about 20% of the world’s adult population [3]. Among processes related to hypertension, angiotensin-I-converting enzyme (ACE) plays an important role in the regulation of blood pressure. ACE is a dipeptidyl carboxypeptidase (EC. 3.4.15.1) and was originally isolated from horse blood [4]. It plays a crucial role in the regulation of blood pressure as it promotes the conversion of angiotensin-I to the potent vasoconstrictor angiotensin-II as well as inactivates the vasodilator bradykinin, which has a depressor action in the renin-angiotensin system. This potent vasoconstrictor is also involved in the release of a Na-retaining steroid, aldosterone from the adrenal cortex, which has a tendency to increase blood pressure [5].

Inhibition of ACE is considered to be a useful therapeutic approach in the treatment of hypertension. Therefore, in the development of drugs to control high blood pressure, ACE inhibition has become an important activity. Many studies have been attempted in the synthesis of ACE inhibitors such as captopril, enalapril, alcacepril and lisinopril, which are currently used in the treatment of essential hypertension and heart failure in humans [6]. However, these synthetic drugs are believed to have certain side effects such as cough, taste disturbances, skin rashes or angioneurotic edema all of which might be intrinsically linked to synthetic ACE inhibitors [7]. Therefore, the research and development to find safer, innovative, and economical ACE inhibitors is necessary for the prevention and remedy of hypertension [8,9]. Many research groups have combed for novel ACE inhibitors from natural products [10], microbial sources [11] and food proteins [12].

Marine organisms are rich sources of structurally diverse bioactive compounds. Hence, a great interest has been developed nowadays to isolate bioactive compounds, which act as ACE inhibitors from marine resources because of their numerous health beneficial effects. According to the researches, it has reported that marine-derived bioactive peptides, chitooligosaccharide derivatives (COS) and phlorotannins have potent ACE inhibitory activity. This review discusses the above three groups of marine-derived ACE inhibitors and their inhibitory properties, emphasizing their potential application as future pharmaceuticals to prevent hypertension.

## 2. Marine-derived ACE Inhibitors and Their Antihypertensive Activity

### 2.1. Bioactive peptides

Components of proteins in food are containing sequences of bioactive peptides, which could exert a physiological effect in the body. These short chains of amino acids are inactive within the sequence of the parent protein, but can be released during gastrointestinal digestion, food processing, or fermentation. Marine-derived bioactive peptides have been obtained widely by enzymatic hydrolysis of marine proteins [13–17]. In fermented marine food sauces such as blue mussel sauce and oyster sauce, enzymatic hydrolysis has already been done by microorganisms, and bioactive peptides can be purified without further hydrolysis [18,19]. In addition, several bioactive peptides have been isolated from marine processing by-products or wastes [20–23]. Marine by-products generated during marine food processing, have low commercial value, but the detection of *in vitro* ACE inhibitory activity in these products might provide significant environmental and cost benefits. Marine-derived bioactive peptides have been shown to possess many physiological functions, including antihypertensive or ACE inhibition [24], antioxidant [25,26], anticoagulant [27,28], and antimicrobial [29,30] activities. Moreover, some of these bioactive peptides have identified to possess nutraceutical potentials that are beneficial in human health promotion [31] and recently the possible roles of food-derived bioactive peptides in reducing the risk of cardiovascular diseases has been shown [32].

Marine-derived antihypertensive peptides have shown potent ACE inhibitory activities (Table 1). The potency of these marine-derived peptides to inhibit ACE activity has been expressed as an IC_50_ value, which is the ACE inhibitor concentration, leading to 50% inhibition of ACE activity. Moreover, the inhibition modes of ACE-catalyzed hydrolysis of these antihypertensive peptides have been determined by Lineweaver-Burk plots.

According to Lineweaver-Burk plot studies, competitive ACE inhibitory peptides have most frequently reported [18,36,40]. These inhibitors can bind to the active site to block it or to the inhibitor binding site that is remote from the active site to alter the enzyme conformation such that the substrate no longer binds to the active sites. Moreover, tryptophan, tyrosine, proline or phenylalanine at the C-terminal and branched-chain aliphatic amino acids at the N-terminal is suitable for peptides to act as competitive inhibitors by binding with ACE [51]. In addition, a non-competitive mechanism has also been observed in some peptides [35,52] and this means that the peptide can combine with an enzyme molecule to produce a dead-end complex, regardless of whether a substrate molecule is bound or not. The hydrophobicity of the N-terminus, which is one of the common features of ACE inhibitory peptides, may contribute to the inhibitory activity [53]. ACE inhibitory peptides are generally short chain peptides, often carrying polar amino acid residues like proline. Furthermore, structure-activity relationships among various peptide inhibitors of ACE indicate that binding to ACE is strongly influenced by the C-terminal tripeptide sequence of the substrate, and it is suggested that peptides, which contain hydrophobic amino acids at these positions, are potent inhibitors [54].

Numerous *in vivo* studies of marine-derived antihypertensive peptides in spontaneously hypertensive rats have shown potent ACE inhibition activity [35,36,40,50] and their systolic blood pressure has reduced significantly after oral administration of peptides. According to Lee *et al*. [36], a single oral administration (10 mg/kg of body weight) of peptide has shown a strong suppressive effect on systolic blood pressure of spontaneously hypertensive rats and this antihypertensive activity was similar with captopril, a commercial antihypertensive drug. Furthermore, they have reported that no side effect observed on rats after administration of antihypertensive peptide. In addition, these marine bioactive peptides exhibit antihypertensive activity *in vivo* than *in vitro*. The exact mechanisms underlying this phenomenon have not yet been identified. However, it was suggested that bioactive peptides have higher tissue affinities and are subject to a slower elimination than captopril [45]. The antihypertensive peptide isolated from bonito fish hydrolysate product, has found to be hydrolyzed by ACE to produce a smaller peptide than the initial one, which had 8-fold ACE inhibitory activity compared with the initial peptide [45].

Therefore, marine-derived bioactive peptides have a potential in use as functional ingredients in nutraceuticals and pharmaceuticals due to their effectiveness in both prevention and treatment of hypertension in addition to the nutritive value. Some antihypertensive synthetic commercial drugs are known to produce side effects such as an abnormal elevation of the blood pressure after administration. However, marine-derived bioactive peptides are well tolerated by the body, are not expected to exert any harmful side effects. In addition, cost effective and safe drugs can be produced from marine bioactive peptides, but further studies are needed with clinical trials for these marine-derived antihypertensive peptides.

### 2.2. Chitooligosaccharide derivatives (COS)

Chitin is the second most abundant biopolymer on earth after cellulose and one of the most abundant polysaccharide [55]. It is a glycan of β (1→4)-linked N-acetylglucosamine units and it is widely distributed in crustaceans and insects as the protective exo-skeleton and cell walls of most fungi. Chitin is usually prepared from the shells of crabs and shrimps. Chitosan, a partially deacetylated polymer of N-acetylglucosamine, is prepared by alkaline deacetylation of chitin [56]. Chitooligosaccharides (COS) are chitosan derivatives (polycationic polymers comprised principally of glucosamine units) and can be generated via either chemical or enzymatic hydrolysis of chitosan [57–59]. Recently, COS have been the subject of increased attention in terms of their pharmaceutical and medicinal applications [60], due to their missing toxicity and high solubility as well as their positive physiological effects such as ACE enzyme inhibition [61], antioxidant [62], antimicrobial [63], anticancer [64,65], immuno-stimulant [66], antidiabetic [67], hypocholesterolemic [68], hypoglycemic [69], anti-Alzheimer’s [70], anticoagulant [71] properties and adipogenesis inhibition [72].

Researches on chitosan and its derivative oligomers have identified their potential to inhibit ACE activity. Even though enough studies have not been carried out, it is presumed that COS may have desirable properties to inhibit ACE activity. Recently, marine-derived chitooligosaccharide (COS) derivatives such as hetero-chitooligosaccharides, aminoethyl chitooligosaccharides, chitin derivatives, chitosan trimer oligomers and carboxylated chitooligosaccharides have been reported as potent ACE inhibitors. They are briefly discussed below as well as summarized in Table 2.

Hong *et al.* [61] studied ACE inhibitory activity of different COS and identified that chitosan trimer is more effective in lowering blood pressure compared to other oligomers. Specifically, the trimer has a lower IC_50_ value (0.9 μM) than most of the other molecular weight COS. In addition, Park *et al*. [73] reported the ACE inhibitory activities of different molecular weight COS or hetero-COS. ACE inhibitory activity of hetero-COS was dependent on the degree of deacetylation of chitosan. They have tested nine kinds of hetero-COS derivatives; prepared by 90%, 75% and 50% *N*-deacetylated of crab chitin with 40% NaOH solution and then each chitosan in to hetero-COS derivatives with relatively high molecular masses (5,000–10,000 Dalton), medium molecular masses (1,000–5,000 Dalton) and low molecular masses (below 1,000 Dalton) by ultrafiltration membrane bioreactor system. Among these hetero-COS, 50% deacetylated and medium molecular weight (1,000–5,000 Dalton) hetero-COS has exhibited the highest ACE inhibitory activity with the IC_50_ value of 1.22 mg/mL (Table 2.) and the inhibition pattern is competitive according to the Lineweaver-Burk plots. These findings suggested that molecular weight and degree of deacetylation of COS are important factors for the ACE inhibitory activity.

To develop ACE inhibitory chitin derivatives, chitin with different degrees of deacetylation have been chemically modified by grafting 2-chloroethylamino hydrochloride on to chitin at the C-6 position [74]. Among three chitin derivatives, such as aminoethyl-chitin with 10% deacetylation (AEC), aminoethyl-chitin with 50% deacetylation (AEC50), and aminoethyl chitin with 90% deacetylation (AEC90), the strongest ACE inhibitor is aminoethyl-chitin with 50% deacetylation (IC_50_ value, 0.038 μM) and the inhibition is competitive. Captopril was used as the positive control (IC_50_ value, 0.1 μM) in this study. Moreover, *in vivo* studies have shown that it effectively decreased the systolic blood pressure of spontaneously hypertensive rats in a dose-dependent manner. When comparing with the IC_50_ values of previous studies, these three water-soluble chitin derivatives have superior ACE inhibitory activity than that of chitosan oligosaccharide derivatives and captopril [74].

Furthermore, the structural properties of chitosan also can be improved by chemical modification to obtain higher active COS derivatives. For example, aminoethyl-COS were synthesized by grafting aminoethyl functional group to improve its ACE inhibitory activity [75]. Hydroxyl groups of the pyranose ring structure at different positions are different chemical attractions and hydroxyl group in the C-6 position was successfully replaced by the aminoethyl group because the structure of COS was maintained due to the C-6 hydroxyl group, which shows the highest reactivity for aminoethylation. Here, another advantage they acquired is that the aminoethyl COS derivatives are easily soluble in water. According to Ngo *et al*. [75], ACE inhibitory activity of aminoethyl-COS (AE-COS) has increased more than that of original COS. At the 2.5 μg/mL concentration, COS and aminoethyl-COS exhibited ACE inhibitory activities of 18.6% and 89.3% respectively. Further, a marked dose-dependent inhibitory effect has been reported in both COS and aminoethyl-COS treatment groups.

Similarly, Huang *et al*. [76] have modified COS by carboxylation with –COCH_2_CH_2_COO^−^ groups to obtain specific structural features similar to captopril. ACE inhibitory activity of carboxylated COS enhances its activity with increased substitution degree and competitive inhibition has been observed. Further, improvement in ACE inhibitory activity of carboxylated-COS compared to COS might be due to the electrostatic interactions between positively charged sites of enzymatic and negatively charged carboxyl groups similar to captopril.

In addition to the ACE, it has shown that renin also plays a vital role in the renin-angiotensin system. Renin (or angiotensinogenase), is a rate-limiting enzyme in the renin-angiotensin system. It cleaves plasma angiotensinogen to angiotensin-I, which is further converted by ACE to angiotensin-II. Therefore, the inhibition of renin activity is also an attractive target in hypertension therapy. Park *et al*. [77] reported that COS have greater potential to inhibit renin activity. According to them, 90% deacetylated and medium molecular weight (1,000–5,000 Dalton) COS has the strongest renin inhibition (IC_50_ value, 0.51 mg/mL) and act as a competitive inhibitor. Collectively, it can be suggested that COS derivatives are potent drug candidates for treat hypertension and their therapeutic role in pharmaceutical industry are assured.

### 2.3. Phlorotannins

Phlorotannins are phenolic compounds formed by the polymerization of phloroglucinol or defined as 1,3,5-trihydroxybenzene monomer units and biosynthesized through the acetate-malonate pathway. They are highly hydrophilic components with a wide range of molecular sizes ranging between 126–650,000 Dalton [78]. Marine brown algae and red algae accumulate a variety of phloroglucinol-based polyphenols, as phlorotannins of low, intermediate and high molecular weight containing both phenyl and phenoxy units [79,80]. Among marine algae, *Ecklonia cava*; an edible brown algae is a rich source of phlorotannins than others [81]. In addition to *E. cava*, other marine algae such as *E. stolonifera*, *Hizikia fusiforme*, *Eisenia bicyclis*, and *Eisenia arborea* have been reported for various phlorotannins [81]. Phlorotannins have several health beneficial biological activities including, antioxidant [82], anti-HIV [83], antiproliferative [84], anti-inflammatory [85], radioprotective [86], antidiabetic [87], anti-Alzheimer’s disease (acetyl cholinesterase and butyryl cholinesterase inhibitory) [88], antimicrobial [89] and antihypertensive or ACE inhibitory activities (Table 3.).

The ACE inhibitory activity of ten Korean seaweeds including, five Phaeophyta (E. cava, E. stolonifera, Pelvetia siliqousa, H. fusiforme, and Undaria pinnatifida), four Rhodophyta (Gigartina tenella, Gelidium amansii, Chondria crassicaulis, and Porphyra tenera), and one Chlorophyta (Capsosiphon fulvescens) have screened by Jung et al. [90]. The ethanol extracts of E. stolonifera, E. cava, P. siliquosa, U. pinnatifida, and G. tenella exhibited significant inhibitory properties against ACE at more than 50% inhibition at a concentration of 163.93 μg/mL. Moreover, they have found that phlorotannins such as eckol, phlorofucofuroeckol A, and dieckol, which derived from E. stolonifera have shown considerable inhibitory activity against ACE. Among them, phlorofucofuroeckol A is the strongest ACE inhibitor with an IC_50_ value of 12.74 μM. Although current knowledge of relationship between the structure and activity of the active phlorotannins is limited, a closed ring dibenzo-1,4-dioxin moiety may be crucial to the above ACE inhibitory effect. In addition, the ACE inhibitory activity may depend on the degree of polymerization of phlorotannin derivatives.

Polyphenolic compounds inhibit ACE activity through sequestration of the enzyme metal factor, Zn^2+^ ion [91]. Therefore, it has been assumed that phlorotannins might form a complex associated with proteins or glycoproteins to inhibit the ACE activity. Athukorala & Jeon [92] have reported that flavourzyme enzymatic digest of E. cava, which contains high content of phlorotannins, is a potent ACE inhibitor, exhibited an IC_50_ of 0.3 μg/ml and captopril, a commercial antihypertensive exhibited an IC_50_ of 0.05 μg/mL. They have hydrolyzed seven marine brown algal species (E. cava, Ishige okamurae, Sargassum fulvellum, Sargassum horneri, Sargassum coreanum, Sargassum thunbergii, and *Scytosiphon lomentaria*) and analyzed for ACE inhibitory activities. Most of the above algal species showed potent ACE inhibitory activities however, *E. cava* is the most potent ACE inhibitor among them due to its rich content of phlorotannins.

Furthermore, among twenty-six marine red algae, an aqueous extracts at 20 °C of *Lomentaria catenata* and *Lithophyllum okamurae* have reported for potent ACE inhibitory activity with IC_50_ values of 12.21 μg/mL and 13.78 μg/mL, respectively [93]. From the methanol extracts at 20 °C, *Ahnfeltiopsis flabelliformis* possessed the highest ACE inhibitory activity (IC_50_, 13.8 μg/mL).

Marine-derived phlorotannins are valuable bioactive compounds and could be introduced for the preparation of novel pharmaceuticals as well as functional foods, and their selection is also a good approach for the treatment or prevention of hypertension.

## 3. Conclusions

Recently, much attention has been paid by consumers towards natural bioactive compounds as functional ingredients and hence it can be suggested that, marine-derived ACE inhibitors are alternative tools that can contribute to consumer’s well-being, by being a part of novel nutraceuticals or pharmaceuticals replacing synthetic drugs. Food bioactive compounds are often effective in promoting health and lead to the reduction of disease risk. Especially, bioactive compounds derived from marine organisms have served as a rich source of health-promoting components. Among them, bioactive peptides, chito-oligosaccharides, and phlorotannins are rich sources of natural health enhancers and this fact implies their potential as a functional ingredient in future nutraceutical and pharmaceutical products. According to presented data, it seems that most IC_50_ values of COS are lower than peptides and phlorotannins, when comparing the ACE inhibitory activity of these marine-derived ACE inhibitors. Until now, most of these ACE inhibitory activities have been observed *in vitro* or in mouse model systems. Therefore, further research studies are needed in order to investigate their activity in human subjects. In conclusion, it can be suggested that marine-derived ACE inhibitors such as bioactive peptides, chitooligosaccharides, and phlorotannins are potential therapeutic candidates for prevent hypertension and their involvement in the future pharmaceuticals are promising.

## Figures and Tables

**Table 1:** ACE inhibitory peptides derived from marine organisms: source, enzyme used for hydrolysis, and IC_50_ value.

Source	Enzyme	IC_50_	Ref.
Blue mussel sauce	natural fermentation	19.34 μg/mL	[19]
Alaska pollack	pronase, flavourzyme	0.21 mg/mL	[33]
Alaska pollack	alcalase, pronase, collagenase	2.6 μM	[34]
Big eye tuna (muscle)	pepsin	21.6 μM	[35]
Big eye tuna (frame)	pepsin	11.28 μM	[36]
Shrimp	protease	0.39 μM	[37]
Shrimp	*Lactobacillus fermentum* enzymes	3.37 mg/mL	[38]
Hard clam	protamex	51 μM	[39]
Sea cucumber	bromelain, alcalase, protease	4.5 μM	[40]
Rotifer	alcalase	9.64 μM	[41]
Wakame	pepsin	21 μM	[42]
Microalga	pepsin	29.6 μM	[43]
Yellow fin sole	α-chymotrypsin	22.3 μM	[44]
Bonito	thermolysin	0.32 μM	[45]
Sardine	alkaline protease	0.015 mg/mL	[46]
Oyster	pepsin	66 μM	[47]
Shark	protease	1.45 μM	[48]
Anchovy fish sauce	natural fermentation	22 μM	[49]
Sea bream	alkaline protease	0.57 mg/mL	[50]

**Table 2:** ACE inhibitory COS derived from marine resources: derivatives and IC_50_ values.

COS derivative	IC_50_	Ref.
Chitosan trimer	0.9 μM	[61]
Chitosan oligosaccharides	1.22 mg/mL	[73]
Chitin derivatives		
AEC	0.064 μM	[74]
AEC 50	0.038 μM	[74]
AEC 90	0.103 μM	[74]
Aminoethyl-COS (AE-COS)	0.80 μg/mL	[75]
Captopril^a^	0.1 μM	[74]

a positive control

**Table 3:** ACE inhibitory phlorotannins derived from marine resources: phlorotannin/fraction and IC_50_ value.

Phlorotannin/fraction	IC_50_	Ref.
Phlorofucofuroeckol A	12.74 μM	[89]
Flavourzyme digest fraction of *E. cava*	0.3 μg/mL	[91]
Methanolic extract of *A. flabelliformis*	13.8 μg/mL	[92]
Captopril^a^	0.05 μg/mL	[91]

a positive control

## Abbreviations

ACE: angiotensin-I-converting enzyme
COS: chitooligosaccharides
AE-COS: aminoethyl chitooligosaccharides
